# Research on the Trajectory and Relative Speed of a Single-Sided Chemical Mechanical Polishing Machine

**DOI:** 10.3390/mi16040450

**Published:** 2025-04-10

**Authors:** Guoqing Ye, Zhenqiang Yao

**Affiliations:** School of Mechanical Engineering, Shanghai Jiao Tong University, Shanghai 200240, China; yeguoqing@sjtu.edu.cn

**Keywords:** trajectory, relative speed, chemical mechanical polishing (CMP), single-sided polishing machine, silicon wafer

## Abstract

This study establishes a bidirectional kinematic analysis framework for single-sided chemical mechanical polishing systems through innovative coordinate transformation synergies (rotational and translational). To address three critical gaps in existing research, interaction dynamics for both pad–wafer and abrasive–wafer interfaces are systematically derived via 5-inch silicon wafers. Key advancements include (1) the development of closed-form trajectory equations for resolving multibody tribological interactions, (2) vector-based relative velocity quantification with 17 × 17 grid 3D visualization, and (3) first-principle parametric mapping of velocity nonuniformity (NUV = 0–0.42) across 0–80 rpm operational regimes. Numerical simulations reveal two fundamental regimes: near-unity rotational speed ratios (ω_P_/ω_C_ = [0.95, 1) and (1, 1.05]) generate optimal spiral trajectories that achieve 95% surface coverage, whereas integer multiples produce stable relative velocities (1.75 m/s at 60 rpm). Experimental validation demonstrated 0.3 μm/min removal rates with <1 μm nonuniformity under optimized conditions, which was attributable to velocity stabilization effects. The methodology exhibits inherent extensibility to high-speed operations (>80 rpm) and alternative polishing configurations through coordinate transformation adaptability. This work provides a systematic derivation protocol for abrasive trajectory analysis, a visualization paradigm for velocity optimization, and quantitative guidelines for precision process control—advancing beyond current empirical approaches in surface finishing technology.

## 1. Introduction

Chemical mechanical polishing (CMP) is an important surface processing technology in semiconductor silicon wafer processing [1]. This is a polishing removal process [2] in which chemical corrosion and mechanical removal are combined under the action of an alkaline polishing slurry suspension [3,4,5,6]. Two stages of combined polishing, rough polishing with a polyurethane polishing pad [7,8,9] and fine polishing with a velvet cloth, are generally divided into rough polishing and fine polishing. In the rough polishing process, the thickness and thickness uniformity of the silicon wafer are mainly controlled. The surface roughness of silicon wafers can reach Ra0.6 nm after rough polishing and then be improved to Ra0.2 nm after precision polishing. The mirror effect is also called mirror polishing to meet the requirements of the downstream process [10].

To control the removal uniformity of the polishing process, many scholars generally use the Preston equation MRR = KPV [11] as the basic equation for removal, where MRR is the material removal rate (MRR), K is the Preston constant, P is the pressure applied to the silicon wafer, and V is the relative speed of the interface. The relative speed of the abrasive particles on the silicon wafer and the polishing pad directly affects the removal rate [12]. However, for single-sided chemical mechanical polishing machines, owing to the eccentric arrangement of the polishing head relative to the polishing plate, different speed ratios can cause dynamic changes in the relative motion trajectory and relative velocity. Therefore, it is necessary to study the motion law within the working speed range and within a larger range. As the polishing pads are bonded to the metal polishing plate together, the polishing plate in this paper refers to the combination of a metal plate and a polishing pad, and the carriers are two independently driven movements. Because the motion trajectory [13,14] and relative speed of each point dynamically change at different speed ratios during processing, some researchers have studied the motion trajectory, relative velocity, and nonuniformity velocity of single-sided polishing machines [15,16,17,18,19,20,21,22]. However, there are three limitations in the current literature, as follows:

(1) The literature only has a one-way abrasive grain trajectory or lacks a detailed mathematical derivation process, which makes it difficult to systematically understand the characteristics of the whole motion in research and engineering applications. Therefore, in this work, motion trajectory parameter equations for silicon wafers to polishing pads and abrasives to silicon wafers of a single-sided polishing machine are constructed and simulated via the rotation and translation coordinate system method.
(2) The literature lacks an intuitive graphical representation of the relative velocity, so this work uses the method of subtracting the velocity vector of the polishing plate and the carrier to derive a mathematical expression of the relative velocity; on this basis, the relative velocity is calculated and graphically simulated.
(3) The literature lacks a complete visual representation of the nonuniformity velocity (NUV) of the whole motion velocity range, so the relative velocity of the polishing plate and the carrier in the speed range of 0–80 rpm is plotted in 3D in a 17 × 17 matrix.

The outstanding achievements of this work are as follows:

(1) The bidirectional motion trajectory equation is established through the synergistic application of rotational and translational coordinate transformations, systematically resolving the multibody interaction dynamics within the tribological interface (wafer–pad–abrasive particle system).
(2) A research method combining relative velocity analysis and silicon wafer polishing experiments is established, which can provide a reference for the relative velocity study of different single-sided polishing machines.
(3) Speed nonuniformity was parameterized via high-resolution parametric 3D mapping (17 × 17 grid configuration), revealing novel distribution patterns across the full operational regime (0–80 rpm).

## 2. Methodology

### 2.1. Introduction of the Chemical Mechanical Polishing Working Image

**Figure 1:**
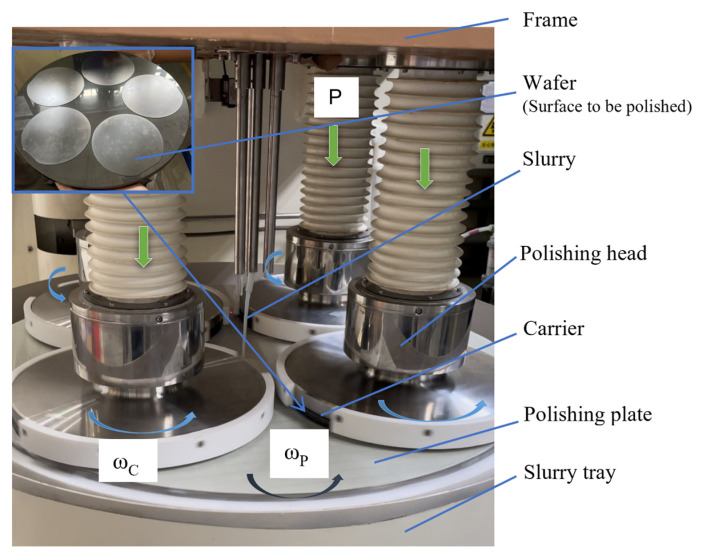
Chemical mechanical polishing working image.

Figure 1 shows a working image of a chemical mechanical polishing machine for processing silicon wafers. The polishing plate is rotated at one process setting speed, and the four sets of polishing heads are fixed on the frame and rotated at the other process setting speed in the same direction. After the wafer is coated with wax and attached to the carrier, it is pressed under the polishing head, which exerts pressure on the carrier. The KOH-containing SiO_2_ abrasive slurry [23,24] was uniformly applied to the surface of the polishing plate, which was covered with a polyurethane pad [25]. CMP achieves atomic-level surface planarization through synergistic chemomechanical interactions: slurry-mediated chemical softening of the silicon surface enables controlled mechanical removal, whereas subsequent exposure to fresh silicon induces passivation layer formation, effectively minimizing subsurface damage [26]. The alkaline slurry based on KOH [27] reacts with silicon substrates according to the following chemical reaction equation:

Si + 2OH^−^ + H_2_O → SiO_3_^2−^ + 2H_2_↑

SiO_3_^2−^ + H_2_O → H_2_ SiO_3_ + 2OH^−^

The material removal research model for chemical mechanical polishing is the Preston equation, which was originally a mathematical model for the study of glass polishing and was later used as a reference equation for CMPs. The Preston model predicts that point P on the workpiece is proportional to the load and relative velocity [28].

(1) $$\frac{dh(x)}{dt}|P=C\frac{dL\left(x\right)}{dA}\frac{ds\left(x\right)}{dt}|P$$

where *h*(*x*) is the grinding depth, *C* is the Preston coefficient, *L*(*x*) is the total load, *A* is the contact area, *s*(*x*) is the sliding distance, and *t* is the process time.

Formula (1) is also often simplified by some scholars to the following equation [29], as shown in Formula (2):

MRR = KPV(2)

where MRR is the material removal rate, K is Preston’s constant, and P is the polishing pressure applied to the effective polishing area. V is the relative speed of the two interfaces.

The established model employs classical wear theory to describe material removal mechanisms in polishing processes. As expressed in Equation (2), the material removal rate (MRR) is directly proportional to both the interfacial pressure within the contact zone and the relative velocity between the workpiece and polishing interface. This relationship originates from the ability of the abrasive particles to achieve a critical indentation depth under the applied normal stress, subsequently inducing shear-driven material detachment through relative motion.

In the context of the chemical mechanical polishing (CMP) investigated herein, localized removal rates at specific wafer locations are positively correlated with instantaneous relative velocity vectors. Our analysis focuses on deriving the kinematic relationship between carrier motion trajectories and resultant velocity fields, establishing quantitative connections between dynamic parameters (ω_P_, ω_C_) and the spatial uniformity of material removal (total thickness variation ≤2 μm).

### 2.2. Total Thickness Variation Inspection of the Silicon Wafer

After chemical mechanical polishing, the silicon wafer was cleaned with ultrasonic waves to remove surface residues, dried, and subjected to morphology detection. One important indicator of removal uniformity is total thickness variation (TTV) [30], which is a noncontact measurement via the 5-point method [31].

**Figure 2:**
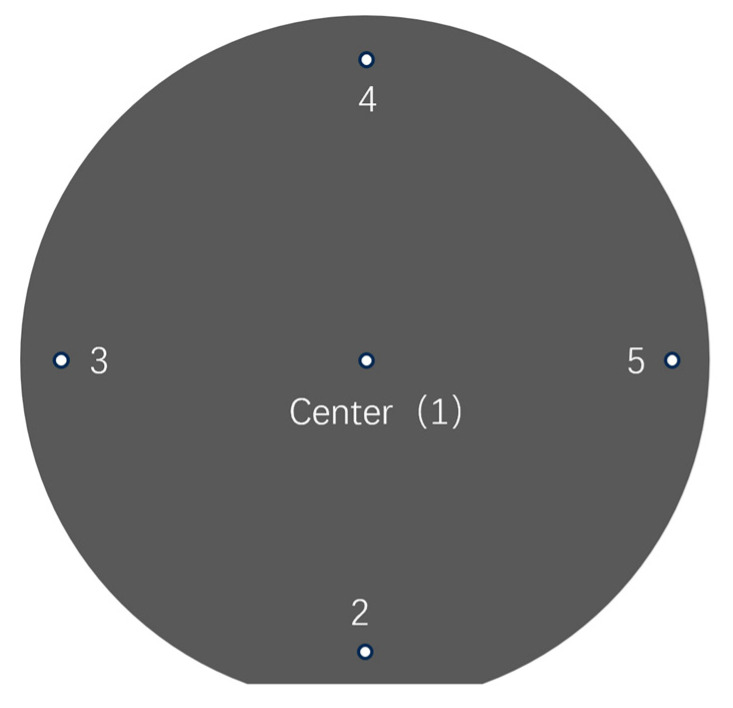
Schematic diagram of the measurement of silicon wafer thickness at 5 points.

As shown in Figure 2, the measuring points of the silicon wafer are center point 1, longitudinal points 2 and 4, and transverse points 3 and 5, and the distance between each point and the edge is 3 mm. These points are measured via noncontact methods, and the thicknesses are *t*_1_, *t*_2_ … *t*_5_.

The expression of the TTV is as follows:

(3) $$TTV=Max\left(t_{1},t_{2},t_{3},t_{4},t_{5}\right)-Min(t_{1},t_{2},t_{3},t_{4},t_{5})$$

Equation (3) indicates that a reduced TTV is correlated with increased thickness uniformity in silicon wafers. Therefore, to obtain high-quality TTV data, it is necessary to control various factors that affect removal uniformity during the polishing process. This study investigated the impact of the polishing plate and polishing head speed on removal uniformity under other optimal processing conditions.

### 2.3. Motion Trajectory of Any Point on the Silicon Wafer Relative to the Polishing Plate (Pad)

**Figure 3:**
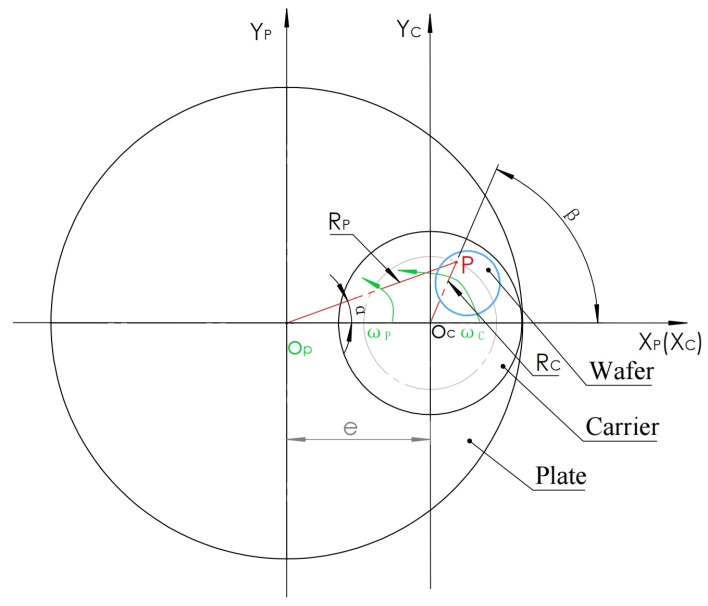
Motion trajectory analysis model diagram.

As shown in Figure 3, the analysis diagram shows the motion relationship diagram composed of the polishing plate, carrier, and wafer. The polishing plate used here is a combination of a metal polishing plate and a polyurethane polishing pad attached to it, which is regarded as one body. The wafer coated with wax is attached to the carrier and is regarded as having no relative motion. The center of the polishing plate is set as the coordinate origin O_P_, the center of the carrier is set as the origin O_C_, the distance between O_P_ and O_C_ is set as e, the direction from O_P_ to the O_C_ is defined as the positive direction of the X_P_ and X_C_ axes, the initial positions X_P_ and X_C_ axes reunite, and the vertical directions with O_P_ and O_C_ axes are the Y_P_ and Y_C_ axes, respectively. Any point on the silicon wafer is set as P, the distance from point P to O_P_ is set as R_P_, the distance from point P to O_C_ is set as R_C_, the angle between the PO_P_ axis and X_P_ axis is set as α, and the angle between the PO_C_ axis and X_C_ axis is set as β. The polishing plate is rotated counterclockwise with O_P_ as the center, and the angular speed is ω_P_. The carrier rotates counterclockwise with O_C_ at the center, and the angular velocity is ω_C_.

**Figure 4:**
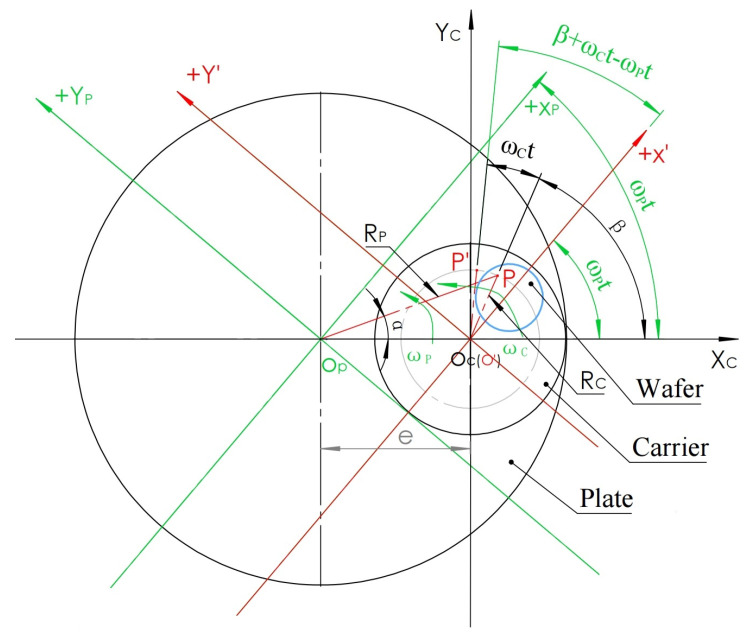
Coordinate system after rotation.

After time t, according to the trigonometric function relationship between the two coordinate systems, the trajectory of the polishing scratches made by point P in the X_P_O_P_Y_P_ coordinate system relative to the polishing plate X_P_O_P_Y_P_ coordinate system is derived as follows:

To find the trace of point P in the X_P_O_P_Y_P_ coordinate system, the X_C_O_C_Y_C_ coordinate system is regarded as fixed, and the X_P_O_P_Y_P_ coordinate system is regarded as rotation. When the polishing plate is rotated counterclockwise at angular velocity ω_P_, the carrier is also rotated counterclockwise. After time t, the coordinate system of the polishing plate rotates counterclockwise around the point O_P_ at the ω_P_t angle, and the P point rotates at the ω_C_t angle around the point O_C_ at the ω_C_t angle, while the X_C_O_C_Y_C_ coordinate system is fixed. To express the coordinate value of the P point in X_P_O_P_Y_P_ after the above two rotation angles, a conversion using an intermediate coordinate system is introduced; this coordinate system is X’O’Y’, as shown in Figure 4.

The X’O’Y’ coordinate system defines O’ to coincide with the point O_C_, while the X’ axis is parallel to and in the same direction as the X_P_ axis rotated by the ω_P_t angle, and the Y’ axis is parallel to and in the same direction as the Y_P_ axis rotated by the ω_P_t angle. In this way, we can first express the P’ coordinate value after the P point rotates the ω_C_t angle around the point O_C_ to the middle coordinate system X’O’Y’ with the origin and then use the coordinate translation principle to express the P’ coordinate system from the middle coordinate system X’O’Y’ to the X_P_O_P_Y_P_ coordinate system with rotation of the ω_P_t angle.

In the intermediate coordinate system X’O’Y’, point P is set as P’ after the rotation of time t from the initial β angle around point O(O’); then, the angle of rotation around the O_C_ is ϖ_C_t, and the plate rotates counterclockwise around point O_P_ in the same direction ϖ_p_t. Since X’ is parallel to the axis of X_P_, according to the principle of equal corresponding angles, the X_C_O_C_(O’)X’ angle is also ϖ_p_t, so the angle X’O’P’ between the X’ axis and O’P’ after rotating P for time t in the X’O’Y’ coordinate system is β + ϖ_c_t − ϖ_p_t (as shown in Figure 4). Therefore, the X’ and Y’ coordinate values of P’ in the X’O’Y’ coordinate system after the time t rotation are expressed in Formula (4).

(4) $$\left[\begin{array}{c} X\\ Y \end{array}\right]=\left[\begin{array}{c} R_{C}cos\left(\beta +\varpi_{C}t-\varpi_{P}t\right)\\ R_{C}sin\left(\beta +\varpi_{C}t-\varpi_{P}t\right) \end{array}\right]$$

Moreover, because the X’O’Y’ coordinate system and the X_P_O_P_Y_P_ coordinate system are parallel coordinate systems, according to the principle of coordinate translation, it is only necessary to obtain the offset value of the coordinate origin O’ relative to point O_P_, which is the X_P_O_P_Y_P_ coordinate of point O’ after rotation, expressed as Equation (5).

(5) $$\left[\begin{array}{c} X\\ Y \end{array}\right]=\left[\begin{array}{c} ecos\varpi_{P}t\\ -esin\varpi_{P}t \end{array}\right]$$

Because the X’O’Y coordinate system is parallel to the X_P_O_P_Y_P_ coordinate system, Formulas (4) and (5) are added to obtain the coordinate value of the P ’point after time t of counterclockwise rotation of the polishing plate at ω_P_ angular velocity and the carrier at ω_C_ angular velocity in the X_P_O_P_Y_P_ coordinate system, as shown in Formula (6).

(6) $$\left\{\begin{array}{c} X_{P}=R_{C}cos\left(\beta +\varpi_{C}t-\varpi_{P}t\right)+ecos\varpi_{P}t\\ Y_{P}=R_{C}sin\left(\beta +\varpi_{C}t-\varpi_{P}t\right)-esin\varpi_{P}t \end{array}\right.$$

In particular, since any point P on the carrier (or silicon wafer) can always be regarded as placing point P in the position of the O_C_X_C_ axis recombination at the starting position, the β value is 0, and Formula (6) can be written as follows:

(7) $$\left\{\begin{array}{c} X_{P}=R_{C}cos\left(\varpi_{C}t-\varpi_{P}t\right)+ecos\varpi_{P}t\\ Y_{P}=R_{C}sin\left(\varpi_{C}t-\varpi_{P}t\right)-esin\varpi_{P}t \end{array}\right.$$

To verify whether the coordinates of parametric Equation (7) are consistent with real motion, a coordinate calculation is carried out before trajectory simulation, and a geometric model is established to measure the coordinate values in the X_P_O_P_Y_P_ coordinate system after rotation. Point P is placed on the X axis when β is 0, and under the conditions of Table 1, the starting point of the trajectory of point P is selected as the distance from the origin of O_P_ coordinate 350, and the O_P_X_P_ and O_C_X_C_ axes coincide, R_C_ = 350e = 72.11.

After calculation and rotation confirmation of the geometric 3D model, the coordinates of the expression of parametric Equation (7) are consistent with the measured values after rotation of the 3D model. The verification procedure is omitted in this article. Therefore, trajectory simulation can be performed.

To visually display the motion trajectory of point P at an O_P_ distance of 350 mm on the silicon wafer, different rotational speed matching conditions, as shown in Table 2, were selected for simulation. The reason for choosing these speed ratio simulations is that the commonly used industrial range is 30–60 rpm, but when a brush wheel is used to clean the polishing pad, the polishing plate may also be below 30 rpm. Therefore, as a study to illustrate the special trajectories generated under multiple relationships, the last three groups were compared with polishing plates at speeds of 20, 10, and 5 rpm.

Table 2 also serves as the speed ratio table for Section 2.4 and Section 2.5 simulations.

**Figure 5:**
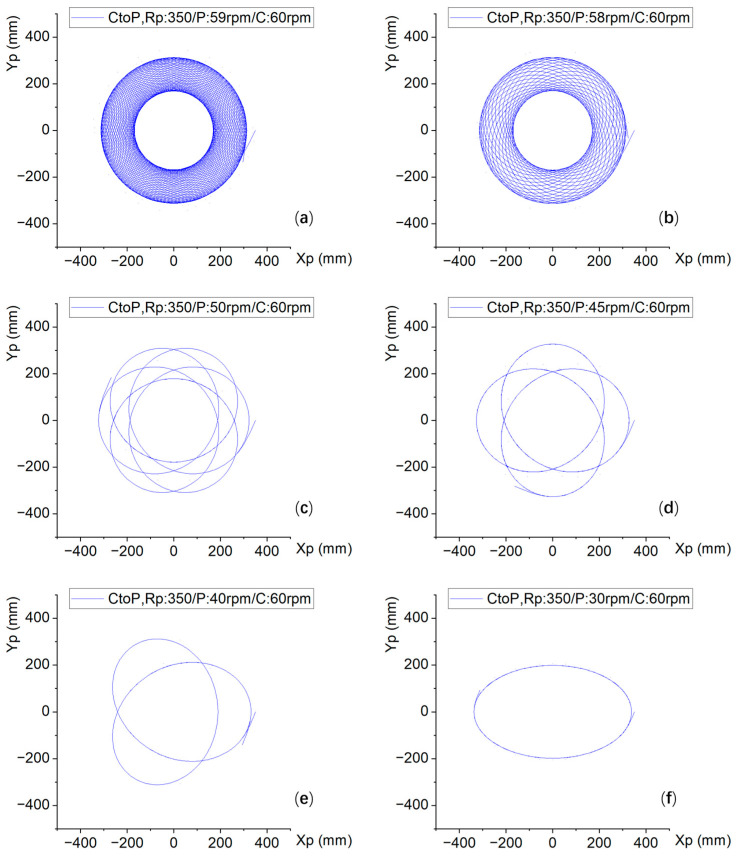

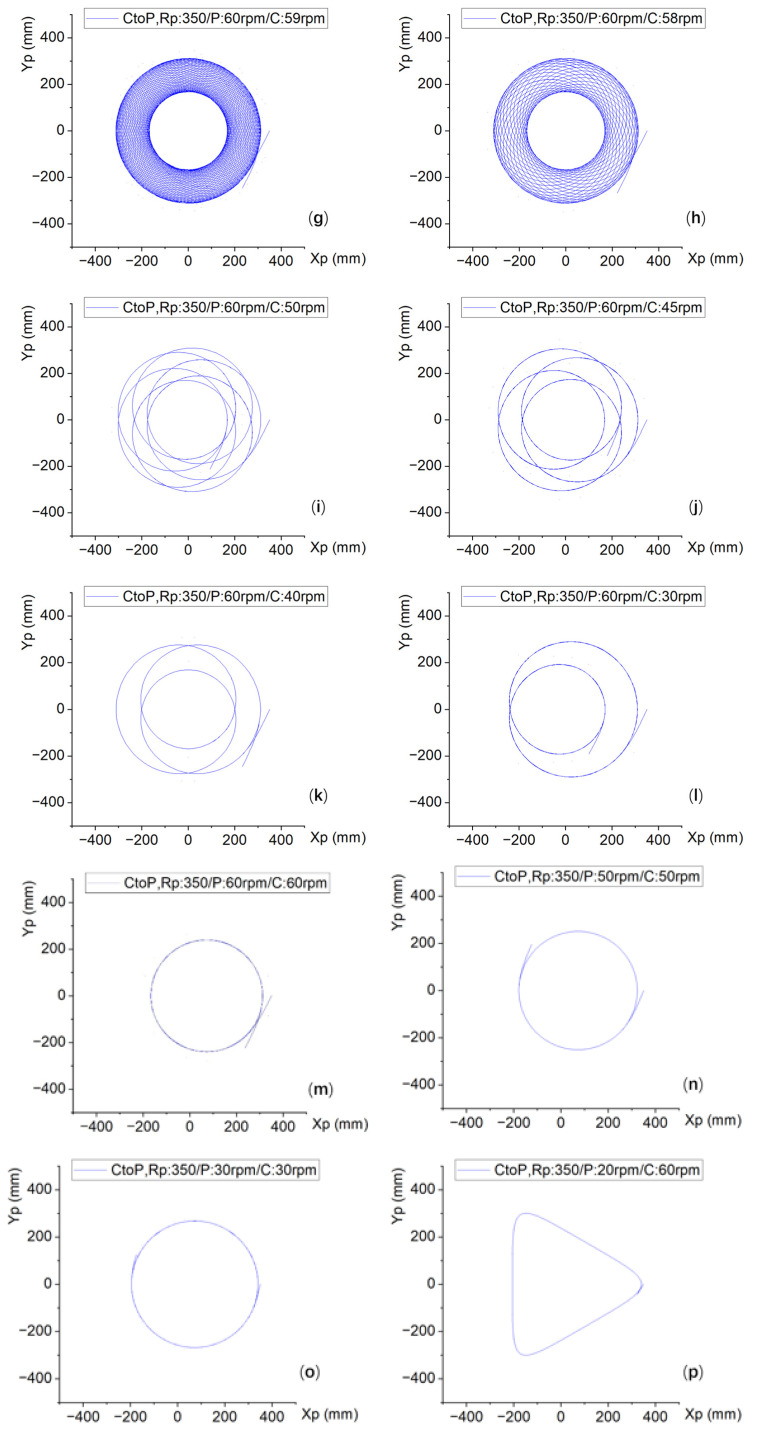

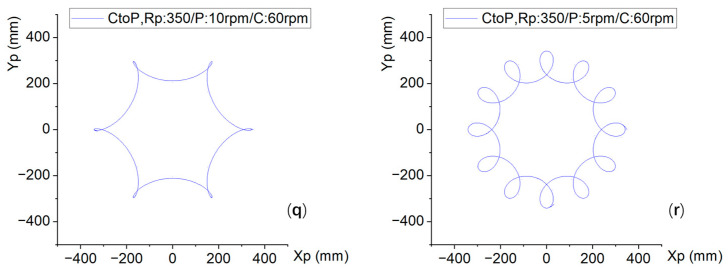
Trajectory diagrams of the carrier-to-polishing plate (CtoP) under various speed ratios at Rp = 350. Plate/carrier speed ratio (rpm). (**a**) 59/60. (**b**) 58/60. (**c**) 50/60. (**d**) 45/60. (**e**) 40/60. (**f**) 30/60. (**g**) 60/59. (**h**) 60/58. (**i**) 60/50. (**j**) 60/45. (**k**) 60/40. (**l**) 60/30. (**m**) 60/60. (**n**) 50/50. (**o**) 30/30. (**p**) 20/60. (**q**) 10/60. (**r**) 5/60.

Figure 5 shows the trajectories under the different combinations of plate and carrier rotation speeds listed in Table 2. In the figure, CtoP represents the trajectory of the carrier (i.e., points on the silicon wafer) on the polishing plate, P represents the plate speed, and C represents the carrier speed. For example, CtoP, R_P_: 350/P: 50 rpm/C: 60 rpm represents the motion trajectory of the carrier on the polishing plate. When Rp is 350, the speed of the polishing plate is 50 rpm, and the speed of the carrier is 60 rpm.

From Figure 5a–f, the trajectories are simulated such that the carrier speed is fixed at 60 rpm and the rotation speed of the polishing plate is gradually reduced. According to the sequence, the trajectories in Figure 5a are the densest. Figure 5b shows that the density is large and that the distribution is uniform. Theoretically, it is possible to contact the relevant area of the polishing pad with a uniform multipath. At the beginning of Figure 5c, the trajectory becomes sparse by a few lines, and by the end of Figure 5c, an elliptical trajectory is formed. This is because at this time, the rotational speed of the polishing plate is 30 rpm, and the carrier rotational speed is 60 rpm, which is an integral multiple of the rotational speed of the carrier.

From Figure 5g–l, the trajectory simulation was carried out so that the rotational speed of the polishing plate was fixed at 60 rpm and the rotational speed of the carrier was gradually reduced. Figure 5g shows the motion trajectory under the conditions of 60 rpm for the polishing plate and 59 rpm for the carrier, revealing the densest routes. Figure 5h shows the motion trajectory at 60 rpm for the polishing plate and 58 rpm for the carrier, revealing a dense and symmetrical trajectory. Its velocity collocation is reversed from Figure 5b, resulting in a similar trajectory. Figure 5i shows the motion trajectory when the rotation speed of the polishing plate is 60 rpm and the rotation speed of the carrier is 50 rpm, which is exactly the opposite of the matching speed of the polishing plate and the carrier in Figure 5c, and the motion trajectory is also relatively similar, with reasonable density and uniformity. Figure 5j shows the motion trajectory when the rotation speed of the polishing plate is 60 rpm and that of the carrier is 45 rpm. This is exactly the opposite of the matching of the rotation speed of the polishing plate and the carrier in Figure 5d, and the density of the trajectory is sparser than that in Figure 5c,i. Figure 5k shows the motion trajectory when the rotation speed of the polishing plate is 60 rpm and the rotation speed of the carrier is 40 rpm, which is exactly the opposite of the matching speed of the polishing plate and the carrier in Figure 5e, and the trajectory density in Figure 5k is greater than that in Figure 5e. Figure 5l shows the motion trajectory when the rotation speed of the polishing plate is 60 rpm and the rotation speed of the carrier is 30 rpm, which is exactly the opposite of the matching rotation speed of the polishing plate and the carrier in Figure 5f. The trajectory in Figure 5l is not elliptical like that in Figure 5f but non-centrosymmetric.

In Figure 5m–o, the velocities of the polishing plate and the carrier are the same: 60 rpm, 50 rpm, and 30 rpm, respectively, and their trajectories appear as the same circles. It can be inferred that the trajectories are circular when the matching speeds are the same.

Figure 5p–r are shown for polishing plate speeds of 20 rpm, 10 rpm, and 5 rpm, respectively, while the carrier speed is the same at 60 rpm. When the carrier speed is 3, 6, or 12 times the integer speed of the polishing plate, there is a special 3-corner shape, similar to the 6-sided shape and 12-circular winding shape, indicating that this shape is related to the corresponding integer multiple. In addition, if the times are opposite multiples, there will be no symmetrical and special graphics, such as triangles, 6 deformations, and 12 circular entanglements, but asymmetric graphics.

From the comparative analysis of the four types of simulation graphs of the 18 speed ratios in Table 2, we observe that trajectory density generally increases with higher relative velocities and closer proximity between objects. Conversely, lower velocities and greater separation distances result in sparser trajectory distributions. Distinct morphological patterns emerge under conditions involving multiple interacting entities. These patterns may exhibit either partial similarity or complete dissimilarity, frequently demonstrating asymmetric configurations. Notably, trajectories assume circular geometries when velocities remain constant across the system.

All trajectories presented in Figure 5 were simulated under fixed radial parameter conditions (R_P_ = 350). Variations in the initial positional parameters yield distinct trajectory patterns across the experimental configurations. However, trajectories consistently maintain circular morphologies under identical rotational velocities, with radius magnitudes showing systematic variation dependent on boundary conditions.

### 2.4. Motion Trajectory at Any Point on the Polishing Plate (Pad) Relative to the Silicon Wafer

To study the rubbing trajectory of the silicon wafer on the carrier by the abrasive particles attached to the polyurethane polishing pad on the metal polishing plate, the reverse mathematical derivation is needed. The range of trajectories that appear is clearly determined by the position and size of the silicon wafer bonded to the carrier.

**Figure 6:**
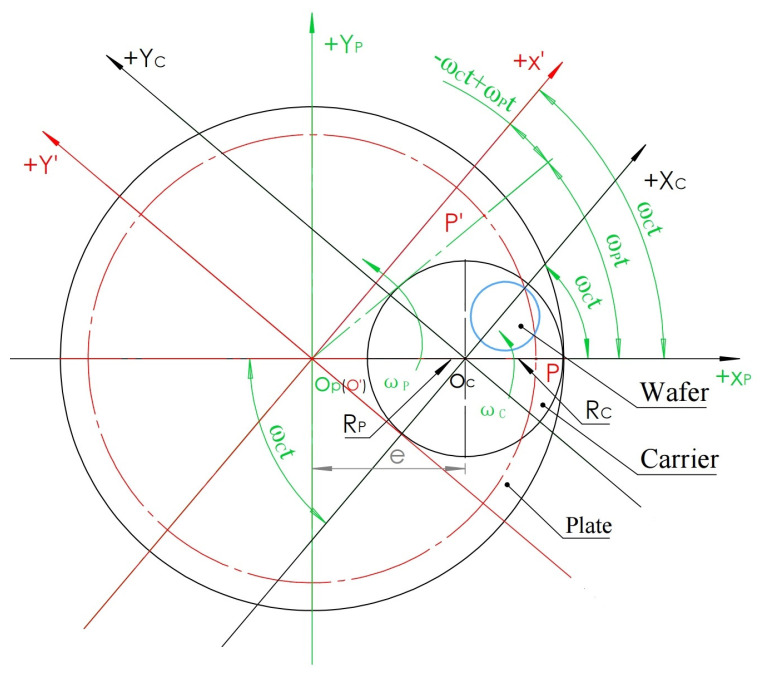
Diagram of the scratching motion of the wafer at one point on the plate.

As shown in Figure 6, since the polishing plate is rotating, the starting point of any point P above can be defined on the X axis. This mathematical model regards the coordinate system X_P_O_P_Y_P_ of the polishing plate as fixed, whereas the carrier coordinate system X_C_O_C_Y_C_ is regarded as a counterclockwise rotation with ω_C_ speed with O_C_ at the center of the circle. When time t passes, the +X_C_ axis rotates the ω_C_t angle counterclockwise. Simultaneously, a point P on the plate rotates counterclockwise around the center of the O_P_ circle at an angle of ω_P_t to arrive at P’. To express the coordinates of P’ relative to the dynamic rotation X_C_O_C_Y_C_ coordinate system, an X’O’Y’ coordinate system used for intermediate calculation is introduced, which is defined as the origin being coincident with O_P_, and X’ is in the same parallel direction as X_C_ after the rotation time t. Y’ is in the same parallel direction as Y_C_ after the rotation time t so that the P’ coordinate value can be expressed in the X’O’Y’ coordinate system first, and then the X’Y’ value can be expressed in the X_C_O_C_Y_C_ coordinate system by the coordinate translation principle.

In Figure 6, in the middle coordinate system X’O’Y’, the point P is set as P’ after the rotation of time t from the coordinate axis Xp around Op(O’); then, the angle of rotation around Op is ϖ_P_t, and the carrier rotates counterclockwise around Oc in the same direction ϖ_C_t. Since X’ is parallel to the Xc axis, according to the principle of equal corresponding angles, the XpOp(O’)X’ angle is also the ω_C_t angle; thus, according to the angle relation, the angle between X’ axis and O’P’ after rotating P in the X’O’Y’ coordinate system for time t is −ϖ_C_t + ϖ_P_t. Therefore, the X’ and Y’ coordinate values of P’ after P rotates at time t in the X’O’Y’ coordinate system are expressed as Formula (8).

(8) $$\left[\begin{array}{c} X\\ Y \end{array}\right]=\left[\begin{array}{c} R_{P}cos\left(-\varpi_{C}t+\varpi_{P}t\right)\\ R_{P}sin\left(-\varpi_{C}t+\varpi_{P}t\right) \end{array}\right]$$

Because the X’O’Y’ coordinate system and the X_C_O_C_Y_C_ coordinate system after rotation at time t are parallel coordinate systems, according to the principle of coordinate translation, it is only necessary to obtain the offset value of the coordinate origin O’ relative to the point O_P_. Since ∠X_C_O_C_X_P_ and ϖ_C_t are opposite angles, they are equal. Therefore, this offset value is the coordinate of X_C_O_C_Y_C_ of point O’ after rotation, expressed as Equation (9):

(9) $$\left[\begin{array}{c} X\\ Y \end{array}\right]=\left[\begin{array}{c} -ecos\varpi_{C}t\\ esin\varpi_{C}t \end{array}\right]$$

Because the X’O’Y’ coordinate system is parallel to the X_C_O_C_Y_C_ coordinate system, Formulas (8) and (9) are added to obtain the coordinate value of the P’ point after time t of counterclockwise rotation of the polishing plate at the ω_P_ angular velocity and the carrier at the ω_C_ angular velocity in the X_C_O_C_Y_C_ coordinate system, as shown in Formula (10).

(10) $$\left\{\begin{array}{c} X_{C}=R_{P}cos\left(-\varpi_{C}t+\varpi_{P}t\right)-ecos\varpi_{C}t\\ Y_{C}=R_{P}sin\left(-\varpi_{C}t+\varpi_{P}t\right)+esin\varpi_{C}t \end{array}\right.$$

Still using the parameters in Table 1, point P, which is 380 away from the O_P_ and re-conjuncts with the initial O_P_X_P_ and O_C_X_C_ axes, is selected to calculate the parameter coordinates of Equation (10) and verify the rotation of the geometric model. The verification results are consistent, and the process is omitted. After verification, the trajectory simulation was carried out under the velocity collocation shown in Table 2.

**Figure 7:**
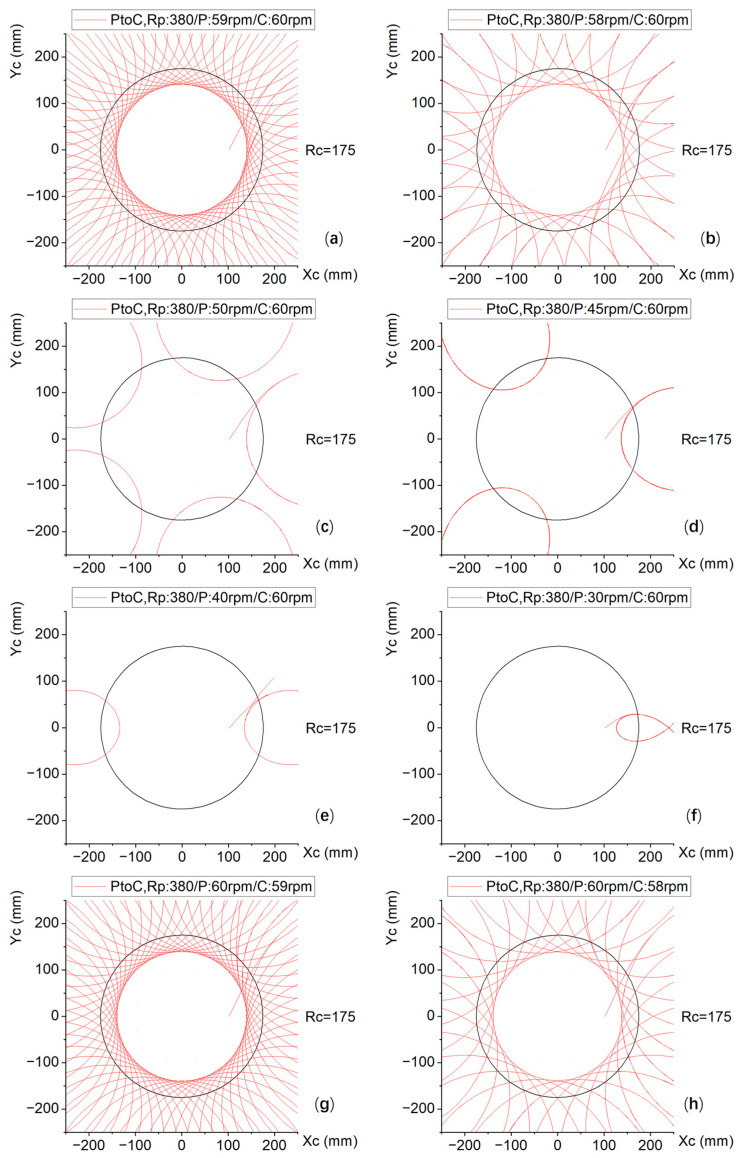

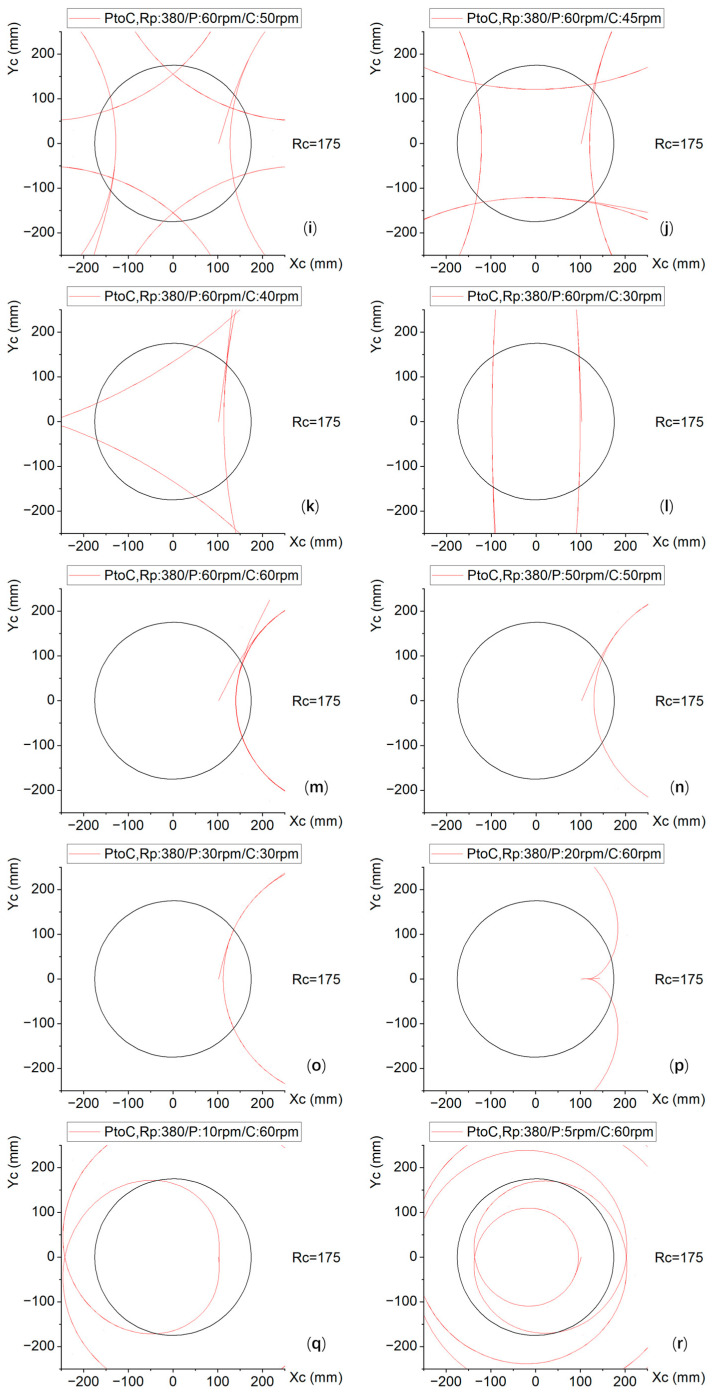
Trajectory diagrams of the polishing plate-to-carrier (PtoC) under various speed ratios at Rp = 380. Plate/carrier speed ratio (rpm). (**a**) 59/60. (**b**) 58/60. (**c**) 50/60. (**d**) 45/60. (**e**) 40/60. (**f**) 30/60. (**g**) 60/59. (**h**) 60/58. (**i**) 60/50. (**j**) 60/45. (**k**) 60/40. (**l**) 60/30. (**m**) 60/60. (**n**) 50/50. (**o**) 30/30. (**p**) 20/60. (**q**) 10/60. (**r**) 5/60.

To visualize the scratching trajectory of the abrasive particles on the silicon wafer, corresponding to the speed ratio in Table 2, trajectory simulation was carried out within the carrier value range of R_C_ = 175, as shown in Figure 7.

Figure 7 shows that the position of a pick on the polishing plate (polishing pad) is the track of R_P_ = 380 particle points across the silicon wafer. The black circle in each figure is the carrier range of R_C_ = 175; there may be a silicon wafer inside, and the outside of the circle is not within the value range.

From Figure 7a–f, the carrier speed remains constant at 60 rpm, whereas the rotational speed of the polishing plate gradually decreases from 59 rpm to 30 rpm. The graph shows that the closer the rotational speed of the polishing plate is to the rotational speed of the carrier, the denser the trajectory; otherwise, the trajectory becomes sparser.

From Figure 7g–l, the rotational speed of the polishing plate remains constant at 60 rpm, whereas the carrier rotational speed gradually decreases from 59 rpm to 30 rpm. The graph shows that the closer the rotational speed of the carrier is to the rotational speed of the polishing plate, the denser the trajectory, and vice versa.

Figure 7m–o show the cases in which the rotation speeds of the plate and the carrier are equal, i.e., 60 rpm, 50 rpm, and 30 rpm, respectively, and the trajectory is a circular section, indicating complete overlap.

Figure 7p–r show that the rotation speeds of the polishing plate are 20 rpm, 10 rpm, and 5 rpm, respectively, whereas the rotation speed of the carrier is 60 rpm. The rotation speed of the carrier is an integer multiple of 3, 6, and 12 times that of the polishing plate, but there is no special trajectory.

Through systematic simulation of abrasive particle trajectories across 18 distinct speed ratio configurations (as specified in Table 2), three principal observations emerge:

Narrow speed ratio differentials (ΔSR < 5%) yield densely clustered trajectory patterns; significant speed ratio disparities (ΔSR > 25%) produce markedly sparse trajectory distributions; and identical speed ratios (ΔSR = 0%) generate singular circular arc trajectories.

The pronounced dispersion in trajectory characteristics across speed ratio variations demonstrates strong parametric sensitivity, with trajectory morphology showing a direct correlation with the speed ratio differential magnitude.

### 2.5. Relative Speeds of the Plate and Carrier at Different Speed Ratios

In Section 2.3, mathematical formulas are derived and simulated for the motion trajectory of the relative polishing plate at one point on the silicon wafer; in Section 2.4, a mathematical formula is derived, and a trajectory simulation is performed for the scratch track of the silicon wafer at one point on the polishing plate. Now, the relative speed of different polishing plates and carrier speeds is analyzed and studied because the relative speed affects the removal rate, which is very important to analyze.

Using the coordinate system defined in Figure 3, the speed of OpXpYp located at point P in the illustrated position relative to the coordinate system of the polishing plate is v_P_, and since the speed direction at this time is opposite to the direction of the OpXp axis, the direction is a negative sign.

(11) $$\overset{⃑}{v_{P}}=R_{P}\overset{⃑}{\varpi_{P}}=(-R_{P}\varpi_{P}cos\alpha ,R_{P}\varpi_{P}sin\alpha )$$

Let the velocity of point P relative to the carrier coordinate system O_C_X_C_Y_C_ be the linear velocity v_C_; since the velocity direction at this time is opposite to the direction of the O_P_X_P_ axis, the direction is a negative sign; then,

(12) $$\overset{⃑}{v_{C}}=R_{C}\overset{⃑}{\varpi_{C}}=(-R_{C}\varpi_{C}cos\beta ,R_{C}\varpi_{C}sin\beta )$$

The relative velocity Δv is the difference between the two vectors.

(13) $$\overset{⃑}{\Delta v}=\overset{⃑}{v_{C}}-\overset{⃑}{v_{P}}=(-R_{C}\varpi_{C}cos\beta +R_{P}\varpi_{P}cos\alpha ,R_{c}\varpi_{c}sin\beta -R_{P}\varpi_{P}sin\alpha )$$

The magnitude of the velocity is the magnitude of the $\overset{⃑}{\Delta v}\;$ vector.

(14) $$\begin{array}{c} \left|\overset{⃑}{\Delta v}\right|=\sqrt{{\left(-R_{C}\varpi_{C}cos\beta +R_{P}\varpi_{P}cos\alpha \right)}^{2}+{\left(R_{C}\varpi_{C}sin\beta -R_{P}\varpi_{P}sin\alpha \right)}^{2}}\\ =\sqrt{{R_{C}}^{2}{\varpi_{C}}^{2}+{R_{P}}^{2}{\varpi_{P}}^{2}-2R_{c}\varpi_{c}R_{P}\varpi_{P}(cos\alpha cos\beta +sin\alpha sin\beta )}\\ =\sqrt{{R_{C}}^{2}{\varpi_{C}}^{2}+{R_{P}}^{2}{\varpi_{P}}^{2}-2R_{C}\varpi_{C}R_{P}\varpi_{P}cos(\alpha -\beta )} \end{array}$$

To understand the relationship between the carrier speed and the rotation speed of the polishing plate under the influence of the relative speed $\left|\overset{⃑}{\Delta v}\right|$ , multiple values of m are introduced, where m is the multiple of the rotation speed of the carrier and the polishing plate, that is,

$$m=\frac{\varpi_{C}}{\varpi_{P}}$$

Therefore, if we multiply the square root of (14) by $\frac{\varpi_{P}^{2}}{\varpi_{P}^{2}}$ , that is, by 1 without changing the size, we obtain the expression related to m (15).

(15) $${\left|\overset{⃑}{\Delta v}\right|=\varpi }_{P}\sqrt{{R_{C}}^{2}m^{2}+{R_{P}}^{2}-2R_{C}R_{P}mcos(\alpha -\beta )}$$

It can be concluded from Formula (15) that in the polishing process, under normal circumstances, the polishing plate and the carrier choose different speed ratio formulas, ω_P_ and m are different, and α and β at different positions under the same speed ratio are also different, which leads to different $\left|\overset{⃑}{\Delta v}\right|$ values; that is, the removal rates are not uniform, but a special case can be found from Formula (15). When m = 1, that is, when the speed of the polishing plate and the carrier is the same, it can be further simplified to obtain a highly deterministic expression. We can substitute m = 1 into Equation (15) to obtain Equation (16):

(16) $${\left|\overset{⃑}{\Delta v}\right|=\varpi }_{P}\sqrt{{R_{C}}^{2}+{R_{P}}^{2}-2R_{C}R_{P}cos(\alpha -\beta )}$$

In the triangle O_P_PO_C_, the outer angle of the triangle is equal to the sum of the two nonadjacent inner angles. Therefore, we can obtain the following expression:

$$\beta =\alpha +∠O_{P}PO_{C}\to ∠O_{P}PO_{C}=\beta -\alpha$$

Since the cosine function is even,

(17) $$\cos\left(\alpha -\beta \right)=\cos\left(\beta -\alpha \right)=cos∠O_{P}PO_{C}$$

Equation (18) is obtained by substituting Equation (17) into Equation (16).

(18) $${\left|\overset{⃑}{\Delta v}\right|=\varpi }_{P}\sqrt{{R_{C}}^{2}+{R_{P}}^{2}-2R_{C}R_{P}cos∠O_{P}PO_{C}}$$

In triangle O_P_PO_C_, the center distance O_P_O_C_ = e is defined according to the cosine law as follows:

(19) $$e^{2}={R_{C}}^{2}+{R_{P}}^{2}-2R_{C}R_{P}cos∠O_{P}PO_{C}$$

Substitute (19) into (18) to obtain

(20) $${\left|\overset{⃑}{\Delta v}\right|=\varpi }_{P}e$$

From the above derivation, under the condition of $m=\frac{\varpi_{c}}{\varpi_{p}}=1$ , the relative speed of any point on the silicon wafer in the polishing process relative to the polishing plate is related only to the angular speed of the polishing plate and the center distance e between the polishing plate and the carrier, and has nothing to do with the position on the silicon wafer. In other words, under these conditions, each point on the silicon wafer can obtain a consistent relative wiping speed; that is, the same removal rate can be obtained to achieve uniform removal, meaning a better TTV value can be obtained.

To determine the relative speed operation rule under different combination speeds of the polishing plate and carrier, the speed curve of Equation (16) is generated.

Substitute ${\alpha =\varpi }_{p}t$ and ${\beta =\varpi }_{c}t$ into Equation (14) to obtain

(21) $$\left|\overset{⃑}{\Delta v}\right|=\sqrt{{R_{C}}^{2}{\varpi_{C}}^{2}+{R_{P}}^{2}{\varpi_{P}}^{2}-2R_{C}\varpi_{C}R_{P}\varpi_{P}cos(\varpi_{P}t-\varpi_{C}t)}$$

**Figure 8:**
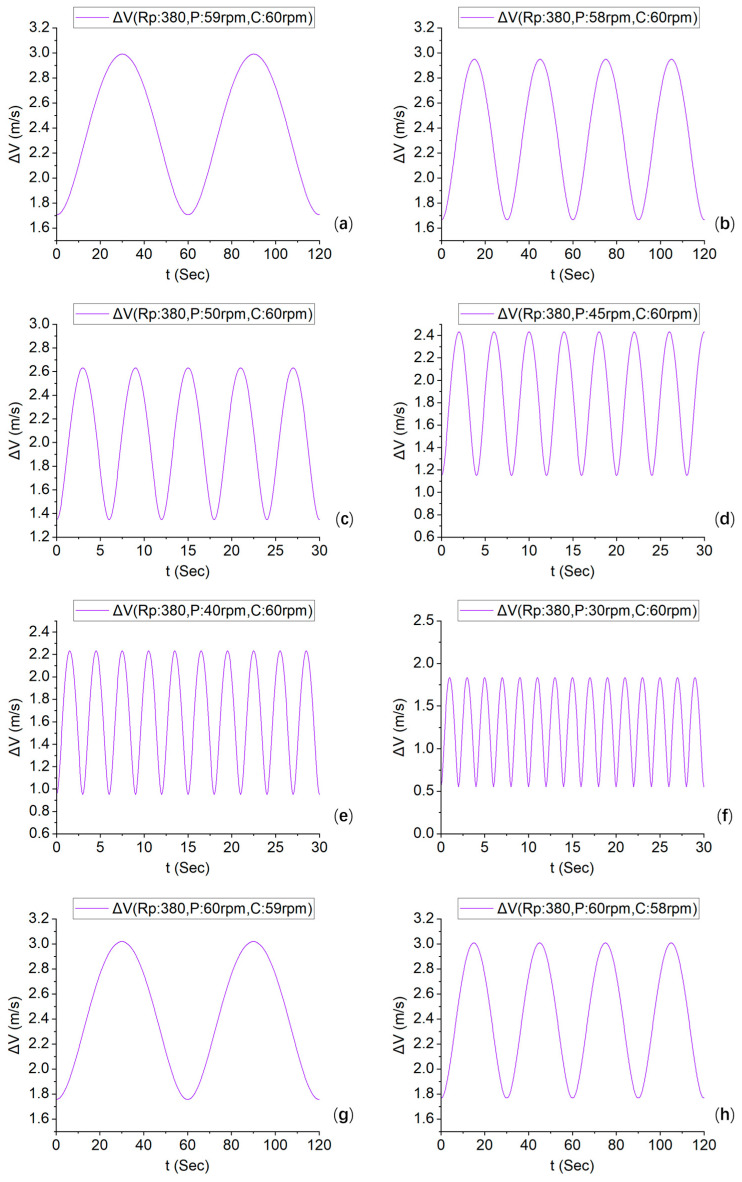

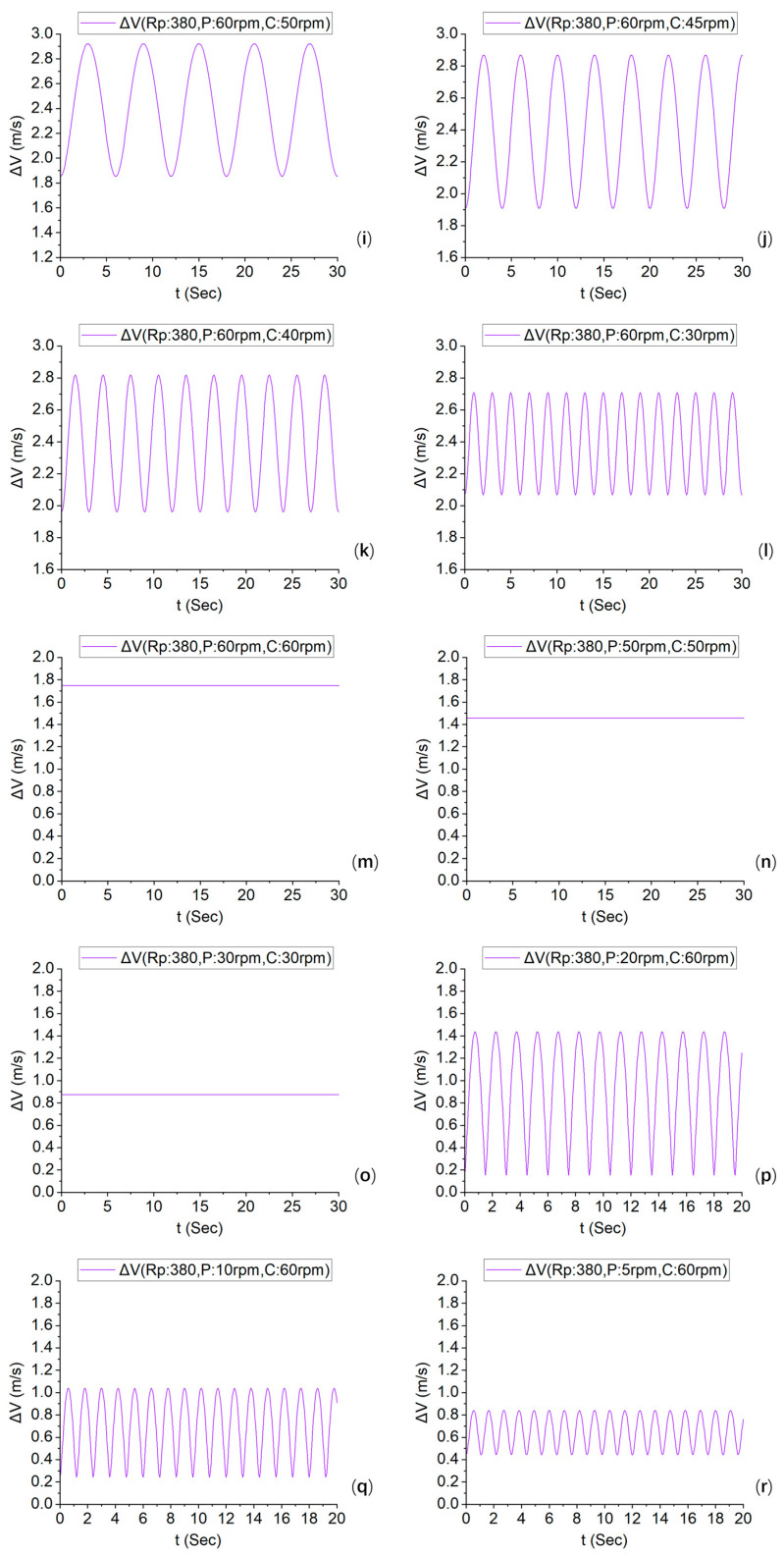
Speed simulation curves with different polishing plates and carrier speeds at Rp = 380. Plate/carrier speed ratio (rpm). (**a**) 59/60. (**b**) 58/60. (**c**) 50/60. (**d**) 45/60. (**e**) 40/60. (**f**) 30/60. (**g**) 60/59. (**h**) 60/58. (**i**) 60/50. (**j**) 60/45. (**k**) 60/40. (**l**) 60/30. (**m**) 60/60. (**n**) 50/50. (**o**) 30/30. (**p**) 20/60. (**q**) 10/60. (**r**) 5/60.

On the basis of Equation (21), the relative velocity between the polishing plate and the carrier was numerically simulated at Rp = 380. The speed ratio parameters for the simulation followed the values provided in Table 2. The resulting relative velocity profile is illustrated in Figure 8.

Figure 8 shows the relative speed of the polishing plate and the carrier. From (a) to (f), the carrier speed remains constant at 60 rpm, whereas the polishing plate speed gradually decreases from 59 rpm to 30 rpm. The relative speed period is gradually reduced from 60 s to 2 s, and the velocity magnitude is also reduced from a minimum of 1.7 m/s to 0.6 m/s.

From (g) to (l), the rotational speed of the polishing plate is maintained at 60 rpm, while the rotational speed of the carrier is gradually reduced from 59 rpm to 30 rpm. The relative speed period gradually decreases from 60 s to 2 s, and the minimum velocity magnitude basically remains between 1.7 m/s and 2.0 m/s.

(m) to (o) show the polishing plate rotation speeds of 60 rpm, 50 rpm, and 30 rpm, respectively, and the carrier rotation speed is the same. In these states, speeds of 1.7 m/s, 1.4 m/s, and 0.9 m/s are maintained in a straight line. Through these three speed ratios, it can be inferred that if the speed of the two is the same, the relative speed is constant.

(p) to (r) show that the carrier speed is fixed at 60 rpm, whereas the rotation speeds of the polishing plate are 20 rpm, 10 rpm, and 5 rpm, and the carrier speeds are 3, 6, and 12 times greater than those of the polishing plate, respectively. At this time, the minimum speed is approximately 0.2 m/s, the period is approximately 2 s, and there is no special curve under the integer ratio speed.

A comparison of the simulation graphs of the relative speed under 18 different speed ratios in Table 2 reveals that, except for the case in which a constant relative speed occurs when the speed ratio is the same, all the others exhibit periodic fluctuations. With respect to cycle duration, when the speed ratios are close to each other, the relative speed shows a longer cycle, and when the speed ratio is higher, there is a significant decrease in the number of cycles. In terms of the relative speed, when the polishing plate speed is high, the relative speed is greater than the inverse proportion of the carrier speed.

To understand the relative speed of different polishing plates and carrier speed ratios more comprehensively, the nonuniformity velocity (NUV) index is used for measurement [32]. The following definition is used:

(22) $$\begin{array}{c} NUV=(1/\bar{v})\frac{1}{n}\sum_{i=1}^{n}\left|v_{i}-\bar{v}\right|\\ {\bar{v}}_{i}=\frac{1}{T}\int_{0}^{T}v_{i}(t)dt \end{array}$$

where ${\bar{v}}_{i}$ is the mean velocity at a given point i along the wafer radius r divided by the total polishing time T, and ${\bar{v}}_{i}$ is the average average velocity at point n (n = 600, 0.2 s interval, calculating the average velocity in the range of 120 s).

**Figure 9:**
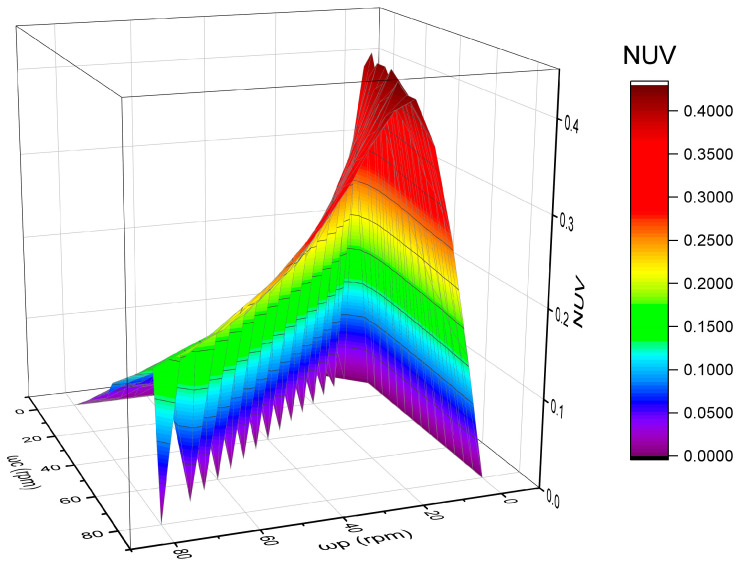
Three-dimensional diagram of the NUV distribution.

The NUV values were calculated when the speed of the polishing plate and the carrier were between 0 and 80 rpm, as the process speed of a typical polishing machine is between 30 and 60 rpm. A point was taken every 5 rpm, the total 17 × 17 = 289 lattice values were calculated, and 3D plotting was carried out, as shown in Figure 9.

Figure 9 shows that the NUV value changes from 0 to 0.4 when the speed of the polishing plate and the carrier changes from 0 to 80 rpm. When the speed of the polishing plate and the carrier is equal, the NUV is 0, indicating that the removal uniformity is mostly due to the speed. However, when a speed difference occurs, the NUV value increases rapidly. Notably, this is the NUV value in the whole theoretical case, and the specific value range is determined by the size and position of the carrier and the size and position of the silicon wafer.

### 2.6. Experimental Procedures

To verify the removal uniformity of silicon wafers at different polishing plate speeds and carrier speeds, the process in Table 3 was used for comparative tests. These parameters, such as the slurry flow rate, load, and polishing time, are based on theoretical research and multiple experiments to obtain optimal conditions. As this paper focuses on the influence of speed on the removal rate and uniformity, these parameters have not been further described.

Under constant experimental conditions, three distinct rotational speed configurations for polishing plates and carriers were systematically investigated: (a) 60 rpm (plate)/60 rpm (carrier), (b) 50 rpm/60 rpm, and (c) 30 rpm/60 rpm. The reason for choosing these speed ratios is to compare their removal rates and uniformity characteristics with the same speed of 60 rpm and the reduced speed of the polishing plate of 50 rpm, as well as a lower speed of 30 rpm, to widen the gap and facilitate the discovery of patterns.

**Figure 10:**
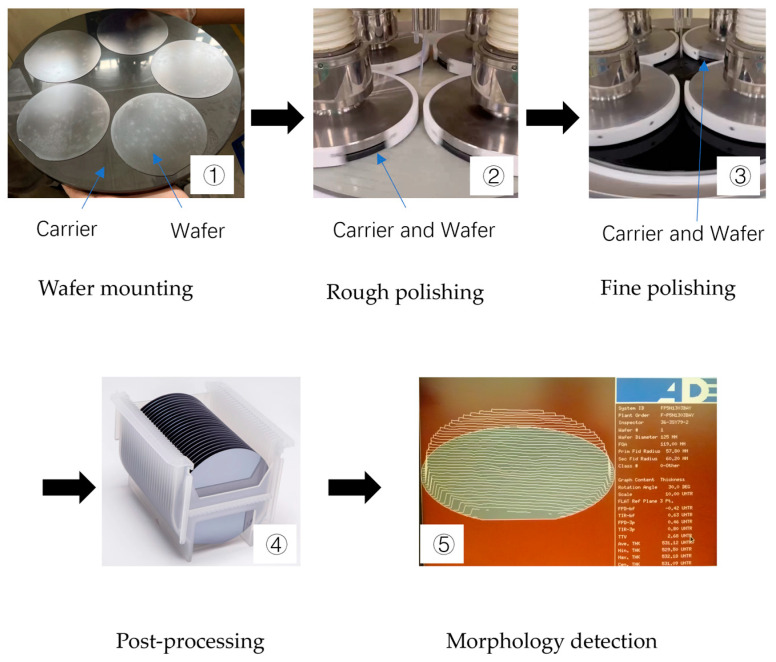
Process diagram of the silicon wafer polishing experiment. ①–⑤ indicate the sequence of experimental stages.

Figure 10 shows a flowchart of the experiment conducted according to the experimental plan in Table 3. The same experimental conditions were used. This experiment aims to change the speed ratio between the polishing plate and the carrier to compare the polishing removal rate and uniformity.

The experimental process comprises five sequential stages:

(1) Wafer mounting: Initial bonding of the silicon wafer to the carrier substrate is achieved through thermal wax deposition.
(2) Rough polishing: A 30 min primary polishing phase is conducted to regulate the total thickness variation (TTV), constituting the critical thickness control stage.
(3) Fine polishing: Subsequent precision processing over approximately 30 min primarily enhances the surface roughness (Ra < 0.2 nm).
(4) Post-processing: Post-polished wafers undergo systematic cleaning in dedicated cassettes through ultrasonic agitation followed by drying with a nitrogen knife.
(5) Morphology detection: Final thickness characterization was performed via noncontact capacitive measurement systems (WaferCheck-8300, ADE Corporation, Westwood, MA, USA) with 0.01 μm resolution, ensuring precise quantification of single-crystal silicon morphology parameters.

## 3. Results and Discussions

**Figure 11:**
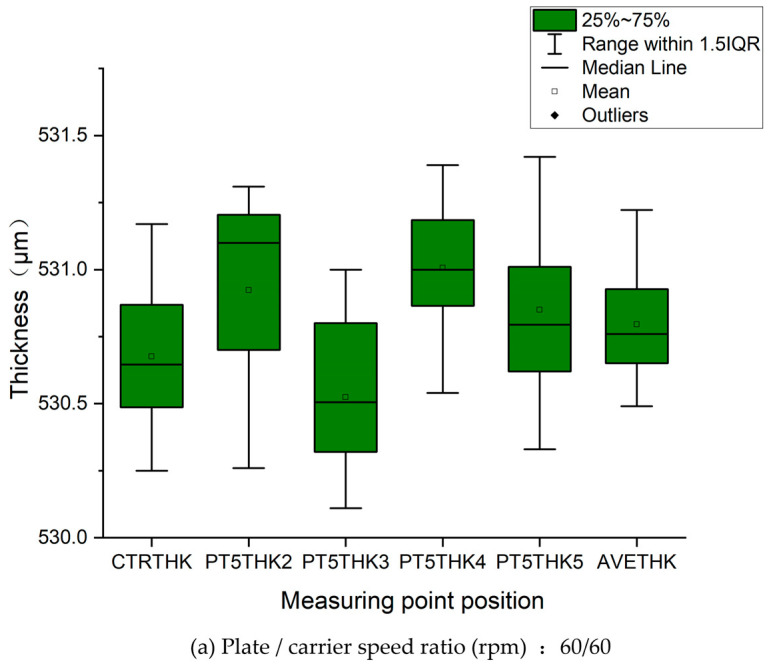

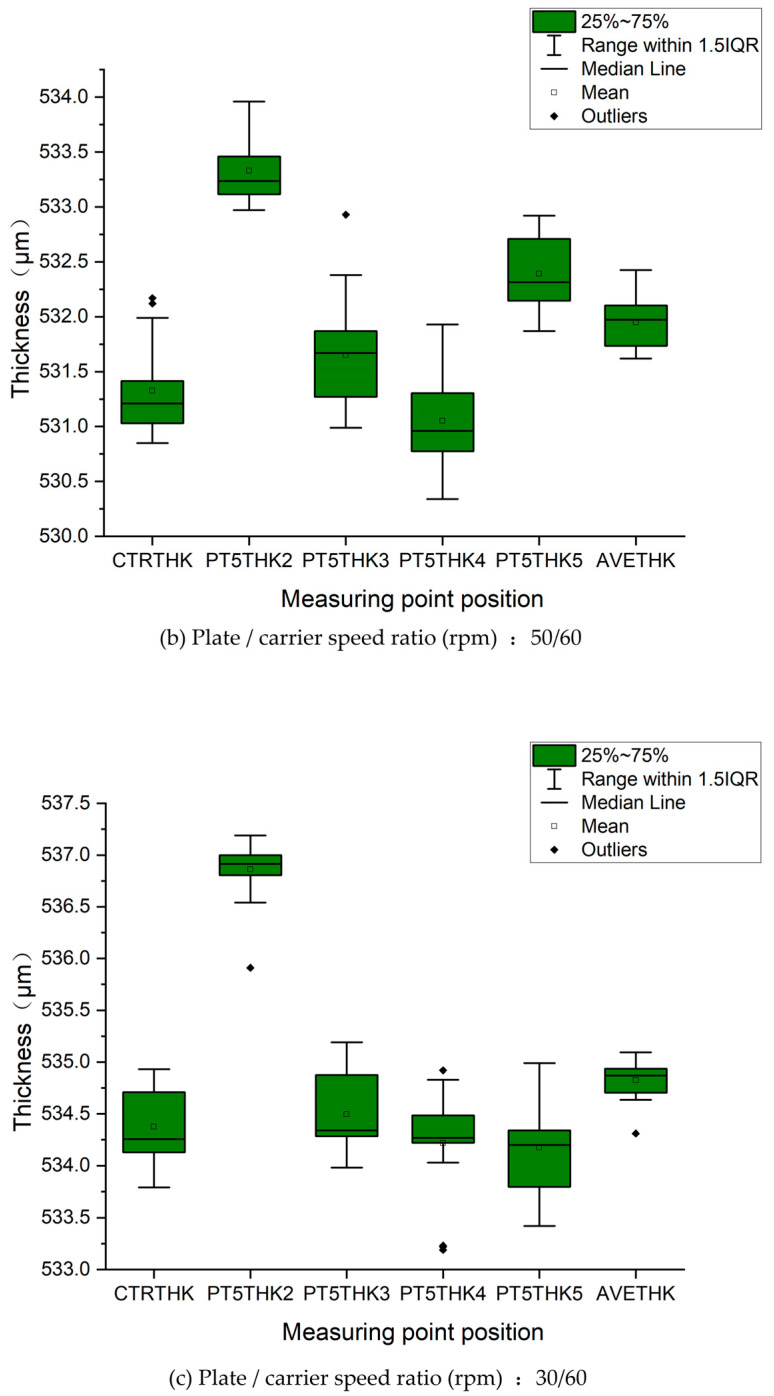
Statistical chart of wafer thickness measurement data. Plate/carrier speed ratio (rpm). (**a**) 60/60. (**b**) 50/60. (**c**) 30/60.

Figure 11 presents the statistical analysis of silicon wafer thickness measurements obtained through the five-point measurement protocol. Following polishing under the experimental conditions specified in Table 3, thickness measurements were systematically recorded via the standardized five-point method. This controlled experiment investigated three distinct rotational speed ratios: (a) 60/60 rpm, (b) 50/60 rpm, and (c) 30/60 rpm. The corresponding measurement locations are schematically illustrated in Figure 2. (Note: AEVTHK denotes the average thickness value, with the initial wafer thickness standardized at 540 μm prior to processing).

Under the same process conditions except for the speed ratio, the polishing plate and carrier speeds in Figure 11a are both 60 rpm, and the thickness data of each point on the silicon wafer are all within the range of 1.5IQR (interquartile range), with a minimum mean of 530.8, indicating the highest removal rate and superior removal uniformity. The polishing plate and carrier speeds in Figure 11b are 50 rpm and 60 rpm, with some points having values outside the 1.5IQR range and a mean of 531.9, indicating a smaller removal rate and inferior removal uniformity compared with (a). The rotation speeds of the polishing plate and carrier in Figure 11c are 30 rpm and 60 rpm, with some point values outside the 1.5IQR range and a mean of 534.8, indicating that the removal rate is the smallest among the three schemes and that the removal uniformity is also inferior.

To further analyze the removal thickness value, the removal thickness is set as THK_i_, the initial condition thickness of the silicon wafer is t_0_, and the measured thickness is t_i_.

Then,

THK_i_ = t_0_ − t_i_

where i is the numbering of multiple test silicon wafers.

**Figure 12:**
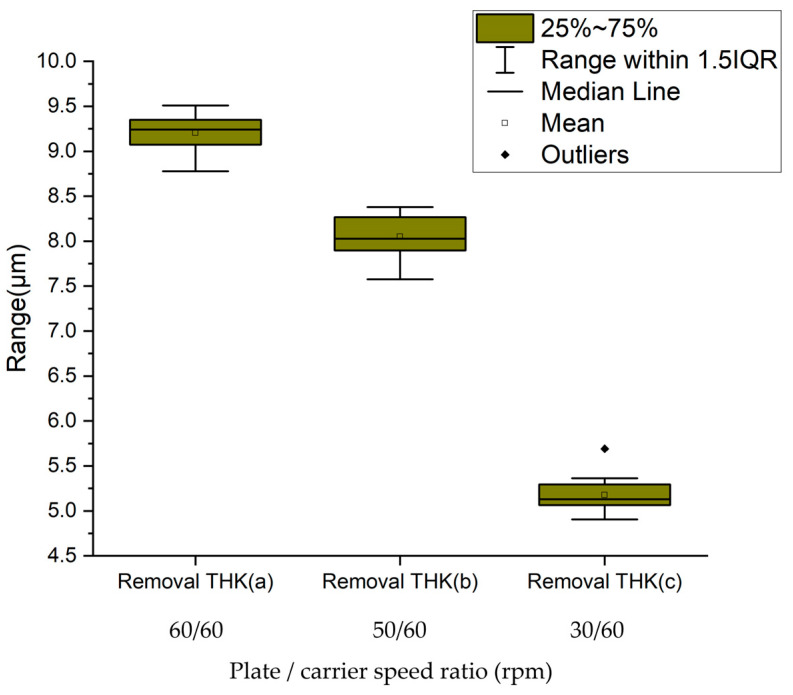
Statistical chart of removal thickness.

Figure 12 quantitatively compares the material removal thickness across three rotational speed ratio configurations: (a) 60/60 rpm, (b) 50/60 rpm, and (c) 30/60 rpm. Statistical analysis revealed significant variation in removal performance, with configuration (a) demonstrating the maximum material removal rate (mean thickness = 9.2 μm), followed by configuration (b) at 8.1 μm. Configuration (c) exhibited the lowest removal efficiency, yielding an average thickness reduction of 5.2 μm.

The quantitative analysis reveals a statistically positive correlation between the material removal thickness and relative velocity magnitude across the three rotational configurations: (a) 60/60 rpm, (b) 50/60 rpm, and (c) 30/60 rpm. As illustrated in Figure 8, configuration (a) maintains a stable relative velocity of 1.7 m/s (Figure 8m), whereas configuration (b) exhibits dynamic velocity fluctuations between 1.4 and 2.6 m/s (Figure 8c). Notably, most of the measured velocity values in configuration (b) exceeded the coverage range of carrier values (see Figure 7c). Configuration (c), corresponding to Figure 8f, demonstrates a reduced velocity range of 0.5–1.8 m/s, with most of the peak velocity out of the coverage range of carrier values (see Figure 7f). Therefore, owing to fluctuations in the speed and actual coverage of the carrier, the removal rate of the polishing plate is 13% lower when the speed ratio is 50/60 than when it is 60/60, and 45.6% lower when the speed ratio is 30/60 than when it is 60/60.

**Figure 13:**
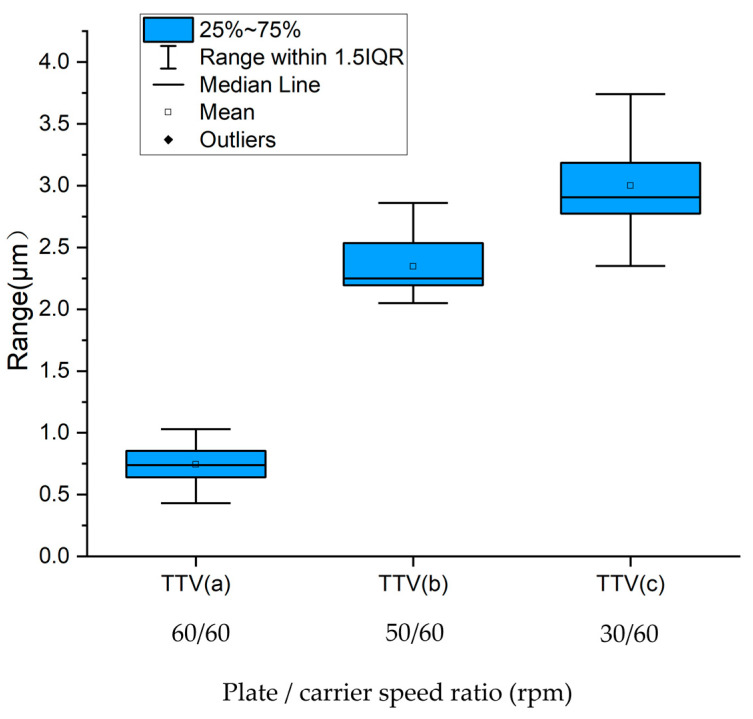
Statistical chart of the TTV.

According to the calculation method of Formula (3), the TTV value of each silicon wafer is calculated, and the corresponding statistical diagram is shown in Figure 13.

Figure 13 compares the total thickness variation (TTV) of silicon wafers processed under three plate/carrier rotational speed configurations: (a) 60/60 rpm (synchronized), (b) 50/60 rpm (asynchronous), and (c) 30/60 rpm (high differential). The synchronized rotation condition (60/60 rpm) yielded the optimal TTV performance, with a mean value of 0.7 μm (δ = 0.025), demonstrating a 76.7% improvement over the worst-case scenario. In contrast, the highly differential configuration (30/60 rpm) produced maximum TTV values averaging 3.0 μm (δ = 0.110), whereas the intermediate asynchronous regime (50/60 rpm) exhibited a transitional TTV of 2.3 μm (δ = 0.049). (δ represents variance).

On the basis of the simulation and experimental results, the discussion is as follows:

(1) The trajectory equation derived on the basis of rotational coordinates, trajectory simulation, and relative velocity simulation can provide a theoretical basis for selecting different speed formulas for single-sided polishing machines. The shapes of different polishing machines at different points are different, and different parameters can be input. Numerical simulations reveal two fundamental regimes; those with near-unity rotational speed ratios (ω_P_/ω_C_ = [0.95, 1) and (1, 1.05]) generate optimal spiral trajectories, achieving 95% surface coverage.
(2) When the speed of the polishing plate and the carrier is an integer multiple, the relative speed is constant, and a relatively uniform removal rate and removal uniformity can be obtained. The higher the relative speed, the faster the removal rate.
(3) Equations for trajectory and relative velocity uniformity can also be applied as a reference for single-sided polishing of other materials, such as SiC. The basic principles are the same.
(4) Due to the sensitivity of the ratio of the polishing plate and speed to the trajectory and relative speed, it is necessary to choose a reasonable speed ratio. Moreover, the accuracy of the deviation between the set speed and the true speed of the polishing machine is also quite important.
(5) Due to the relative velocity fluctuations caused by the different rotational speeds of the polishing plate and the carrier, as well as the limitations of the carrier coverage range, traditional steady-state models have difficulty describing this state. Therefore, an effective velocity integration model can be considered, i.e., the MRR = K P ∫ΔV (t)dt description, where ΔV is the relative velocity, as shown in Equation (14) and Figure 8, which exhibits dynamic variability.

## 4. Conclusions

This study advances the theoretical framework for analyzing motion dynamics in single-sided polishing systems. The key findings are summarized as follows:

(1) Trajectory parameterization and derivation

A bidirectional motion trajectory equation was developed through synergistic rotational and translational coordinate transformations. This methodology resolves multibody interaction dynamics at the tribological interface (pad–abrasive–wafer system) and provides a systematic derivation process absent in prior studies.

(2) Relative velocity analysis

A relative velocity law was established by vectorially subtracting the polishing plate and carrier velocities, accompanied by high-resolution 3D graphical simulations (17 × 17 grid).

This visualization framework overcomes the limitations of text-based velocity descriptions in previous works, offering intuitive insights for optimizing speed combinations.

(3) Parametric nonuniformity mapping

The nonuniformity velocity (NUV) was quantified across the full operational range (0–80 rpm) via parametric 3D mapping. The novel grid-based approach reveals previously undocumented velocity distribution patterns critical for process control.

(4) Methodological extensibility

The proposed coordinate transformation methodology remains valid for high-speed applications (>80 rpm) and adaptable to similar polishing configurations.

The velocity analysis framework provides a template for studying other single-platen polishing systems.

## Figures and Tables

**Table 1:** Motion trajectory simulation parameters.

Dimension	Radius (mm)
Carrier	175
Plate (Pad)	457
Wafer	62.5
e	277.89

**Table 2:** Simulation of the speed ratio between the polishing plate and the carrier.

Number	Rotation Speed of Plate (rpm)	Rotation Speed of Carrier (rpm)
(a)	59	60
(b)	58	60
(c)	50	60
(d)	45	60
(e)	40	60
(f)	30	60
(g)	60	59
(h)	60	58
(i)	60	50
(j)	60	45
(k)	60	40
(l)	60	30
(m)	60	60
(n)	50	50
(o)	30	30
(p)	20	60
(q)	10	60
(r)	5	60

**Table 3:** Experimental conditions.

Component	Configuration
Pad	MH^TM^-S15S (NITTA DuPont INCORPORATE, Osaka, Japan) Diameter: 914 mm
Polishing slurry	AJ-7120 colloided silica, Fujimi Corporation, Kiyosu, Aichi Prefecture, Japan Chemical additive: KOH, PH value of polishing solution: PH11 Flow rate: 2 L/min
Carrier	SiC ceramic (waxing with silicon wafers), 4pcs
workpiece	Material: Silicon wafer Diameter:125 mm, Thickness:540 μm Quantity: 4 × 5 = 20
Applied load	250 N (15 s) → 1050 N (29 min) → 250 N (75 s)
Rotational speed of polishing plate/carrier	(a) 60 rpm/60 rpm (b) 50 rpm/60 rpm (c) 30 rpm/60 rpm

## Data Availability

The original contributions presented in the study are included in the article, further inquiries can be directed to the corresponding author.

## References

[1] Moon Y., Babu S. (2022). 1-Chemical and Physical Mechanisms of Dielectric Chemical Mechanical Polishing (CMP). Advances in Chemical Mechanical Planarization (CMP).

[2] Lu S., Xia J., Yu J., Wang Z. (2024). Investigating the Partial Plastic Formation Mechanism of Typical Scratches on Silicon Wafers Induced by Rogue Particles During Chemical Mechanical Polishing. Mater. Sci. Semicond. Process..

[3] Srivastava M., Singh J., Mishra D.K., Singh R.P. (2022). Review on the Various Strategies Adopted for the Polishing of Silicon Wafer—A Chemical Perspective. Mater. Today Proc..

[4] Datta D., Rai H., Singh S., Srivastava M., Sharma R.K., Gosvami N.N. (2022). Nanoscale Tribological Aspects of Chemical Mechanical Polishing: A Review. Appl. Surf. Sci. Adv..

[5] Pandey K., Sharma A., Singh A.K. (2022). Silicon Wafers; Its Manufacturing Processes and Finishing Techniques: An Overview. Silicon.

[6] Seo J. (2021). A Review on Chemical and Mechanical Phenomena at the Wafer Interface During Chemical Mechanical Planarization. J. Mater. Res..

[7] Cook L., Babu S. (2022). 21-CMP Pads and Their Performance. Advances in Chemical Mechanical Planarization (CMP).

[8] Uneda M., Maeda Y., Ishikawa K., Ichikawa K., Doi T., Yamazaki T., Aida H. (2011). Relationships between Contact Image Analysis Results for Pad Surface Texture and Removal Rate in CMP. J. Electrochem. Soc..

[9] Jeong J., Shin Y., Jeong S., Jeong H. (2025). Characterizing the Contact Evolution through the Combination of Surface Roughness Parameters in Chemical Mechanical Polishing Using a Polyurethane Polishing Pad. Wear.

[10] Zhao D., Lu X. (2013). Chemical Mechanical Polishing: Theory and Experiment. Friction.

[11] Luo Z., Zhang Z., Zhao F., Fan C., Feng J., Zhou H., Meng F., Zhuang X., Wang J. (2024). Advanced Polishing Methods for Atomic-Scale Surfaces: A Review. Mater. Today Sustain..

[12] Zhang J., Jiang Y., Luo H., Yin S. (2021). Prediction of Material Removal Rate in Chemical Mechanical Polishing via Residual Convolutional Neural Network. Control Eng. Pract..

[13] Cheng Z., Gao H., Liu Z., Guo D. (2020). Investigation of the Trajectory Uniformity in Water Dissolution Ultraprecision Continuous Polishing of Large-Sized KDP Crystal. Int. J. Extreme Manuf..

[14] Zhao X., Wang S., Zhang N., Hao Q., Shi F. (2023). High Steepness Aspheric Polishing Trajectory Planning Based on Equal Arc Length Sampling. Proceedings of the AOPC 2023: Optical Design and Manufacturing.

[15] Hocheng H., Tsai H.Y., Tsai M.S. (2000). Effects of Kinematic Variables on Nonuniformity in Chemical Mechanical Planarization. Int. J. Mach. Tools Manuf..

[16] Zhao D., Wang T., He Y., Lu X. (2013). Kinematic Optimization for Chemical Mechanical Polishing Based on Statistical Analysis of Particle Trajectories. IEEE Trans. Semicond. Manuf..

[17] Wu M., Wu Y., Lai Z., Xu Z., Huang H. (2025). Design and Experimental Validation of Non-Trimming Polishing Plate for High Wear-Resistant Workpieces Based on Optimized Motion Trajectory Distribution. Precis. Eng..

[18] Yang K., Huang N., Di H., Zhou P. (2025). Modeling of Surface Microtopography Evolution in Chemical Mechanical Polishing Considering Chemical-Mechanical Synergy. Tribol. Int..

[19] Jiang B., Guan J., Zhao P., Chen Y., Zhang Z. (2024). Effect of Polyoxyethylene-Based Nonionic Surfactants on Chemical–Mechanical Polishing Performance of Monocrystalline Silicon Wafers. Crystals.

[20] Yoshitomi K., Shimada Y., Une A. (2023). Development of High-Speed Rotation Polishing System with Slurry Confinement and Friction-State Control. Int. J. Autom. Technol..

[21] Zheng P., Zhao D., Lu X. (2023). Prediction of Pad Wear Profile and Simulation of Its Influence on Wafer Polishing. Micromachines.

[22] Ma J., Wan H., Peng F., Chen H., Chen C., Chen P., Beri T.H., Chen H., Ren K., Lyu B. (2024). Study on Grain Removal Characteristics and Influencing Factors of Polycrystalline Tungsten during Polishing Process. Precis. Eng..

[23] Irfan H.M., Lee C.-Y., Mazumdar D., Aryanfar Y., Wu W. (2025). Improvement of Material Removal Rate and Within Wafer Non-Uniformity in Chemical Mechanical Polishing Using Computational Fluid Dynamic Modeling. J. Manuf. Mater. Process..

[24] Hwang S., Park J., Kim W. (2024). The Stability Evaluation of Ceria Slurry Using Polymer Dispersants with Varying Contents for Chemical Mechanical Polishing Process. Polymers.

[25] Jeon S., Hong J., Hong S., Kanade C., Park K., Seok H., Kim H., Lee S., Kim T. (2021). Investigation of Abrasive-Free Slurry for Polysilicon Buffing Chemical Mechanical Planarization. Mater. Sci. Semicond. Process..

[26] Huo Y., Niu Y., Sun Z., Li Y., Niu J. (2024). Surface/Subsurface Damage Mechanisms and Inhibition Strategies in Machining of Hard and Brittle Materials: A Systematic Review. Surf. Interfaces.

[27] Bae J.-Y., Han M.-H., Lee S.-J., Kim E.-S., Lee K., Lee G.-S., Park J.-H., Park J.-G. (2022). Silicon Wafer CMP Slurry Using a Hydrolysis Reaction Accelerator with an Amine Functional Group Remarkably Enhances Polishing Rate. Nanomaterials.

[28] Leo J., Tan H., Ma Y., Parab S.M., Huang Y., Wang D., Zhu L., Lam J., Mai Z. (2017). Key Issues for Implementing Smart Polishing in Semiconductor Failure Analysis. J. Appl. Math. Phys..

[29] Li S., Fu J., He Z., Luo Y., Wu S. (2023). Nanomaterials and Equipment for Chemical–Mechanical Polishing of Single-Crystal Sapphire Wafers. Coatings.

[30] Ye G., Yao Z. (2025). Research on Deflection and Stress Analyses and the Improvement of the Removal Uniformity of Silicon in a Single-Sided Polishing Machine Under Pressure. Micromachines.

[31] Liu Y., Tao H., Zhao D., Lu X. (2022). An Investigation on the Total Thickness Variation Control and Optimization in the Wafer Backside Grinding Process. Materials.

[32] Tseng W.T., Chin J.H., Kang L.C. (1999). A Comparative Study on the Roles of Velocity in the Material Removal Rate During Chemical Mechanical Polishing. J. Electrochem. Soc..

