# Correction: Ye, G.; Yao, Z. Research on the Trajectory and Relative Speed of a Single-Sided Chemical Mechanical Polishing Machine. *Micromachines* 2025, *16*, 450

**DOI:** 10.3390/mi17020160

**Published:** 2026-01-26

**Authors:** Guoqing Ye, Zhenqiang Yao

**Affiliations:** School of Mechanical Engineering, Shanghai Jiao Tong University, Shanghai 200240, China

## Error in Figure

In the original publication [1], there was a mistake in Figure 5m as published. C: 70 rpm. The corrected Figure 5m appears below.

The subplot annotation ‘C: 70 rpm’ of the original paper [1] was mistakenly used in Figure 5m, contrary to the actual simulation parameter and contextual evidence documented below. The simulation data (curves in Figure 5m) were generated at 60 rpm, with consistency across the following:

Tabulated speed ratio (60/60) in Table 2

Correction:

‘C: 70 rpm’ → ‘C: 60 rpm’

This stand-alone labeling error had no impact on graphical integrity or conclusions.


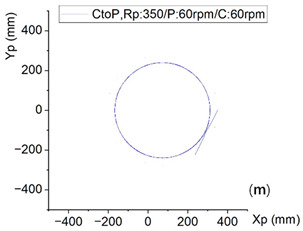


The authors state that the scientific conclusions are unaffected. This correction was approved by the Academic Editor. The original publication has also been updated.

## Text Correction

There was an error in the original publication Equation (10).

**Insert CORRECTED paragraph**

Subscripts in Equation (10) of the original paper [1] contained inconsistent notation:

(1) X_P_/Y_P_ was used, contrary to simulation coordinates X_C_/Y_C_ in Figure 7.

(2) $-ecos\varpi_{P}t$/$+esin\varpi_{P}t$ appeared, inconsistent with $\varpi_{C}$ in Equation (9).

Correction:

(1) X_P_ → X_C_; Y_P_ → Y_C_

This ensures correspondence with simulation coordinates (X_C,_ Y_C_) in Figure 7.

(2) $-ecos\varpi_{P}t$ → $-ecos\varpi_{C}t$

$+esin\varpi_{P}t$ → $+esin\varpi_{C}t$

(10) $$\{\begin{array}{c} X_{C}=R_{P}cos\left(-\varpi_{C}t+\varpi_{P}t\right)-ecos\varpi_{C}t\\ Y_{C}=R_{P}sin\left(-\varpi_{C}t+\varpi_{P}t\right)+esin\varpi_{C}t \end{array}$$

This typesetting error only affected symbolic presentation; the mathematical validity and simulation results remain unchanged.

The authors state that the scientific conclusions are unaffected. This correction was approved by the Academic Editor. The original publication has also been updated.
